# Retracted: The Antioxidant and Starch Hydrolase Inhibitory Activity of Ten Spices in an *In Vitro* Model of Digestion: Bioaccessibility of Anthocyanins and Carotenoids

**DOI:** 10.1155/2016/4142104

**Published:** 2016-09-15

**Authors:** Evidence-Based Complementary and Alternative Medicine

Evidence-Based Complementary and Alternative Medicine has retracted the article titled “The Antioxidant and Starch Hydrolase Inhibitory Activity of Ten Spices in an *In Vitro* Model of Digestion: Bioaccessibility of Anthocyanins and Carotenoids” [1], as the High Performance Liquid Chromatography (HPLC) results were questioned and it was found that the underlying data for this study are not available, and thus the results of the article are questionable.
